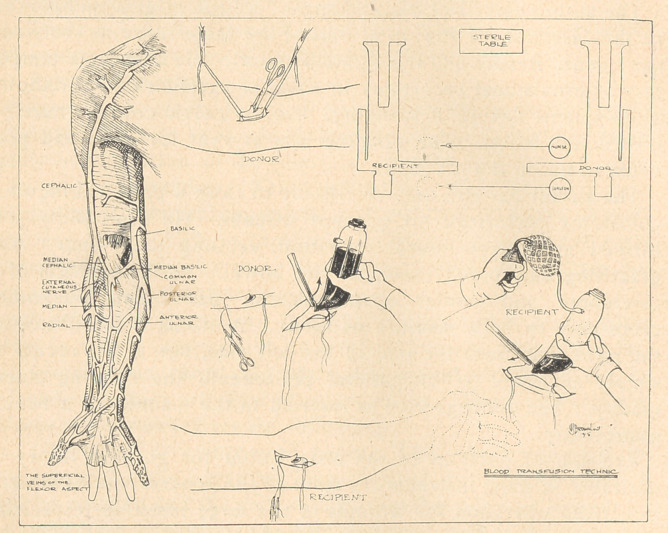# Blood Transfusion at the Front

**Published:** 1918-09

**Authors:** 


***Blood Transfusion at the Front Area.*** By BENJAMIN I. HARRISON, Lieut. M. R. C. Preliminary Note of an article to appear elsewhere.

The author's method of selecting donors and of grouping recipients was that described by Lee. A grouped list of all donors was available at a moment's notice day or night. By way of economy Group 2, 3, and 4 men were used. The indirect methods of transfusion were chosen, a vein of the flexor aspect of the arm, 3 cc. of 1 o/o novocaine being used for infiltration anesthesia. The proximal end of the donor's vein was tied, and the distal end of the recipient's. A ligature loop was placed round the distal end of the donor's vein and the proximal end of the recipient's. Traction by means of a hemostat closed the veins.

With regard to diagnosis, the author remarks that in hemorrhage, the patient is generally restless and anxious, or worried about his condition. A shock-patient on the other hand is listless and apathetic. In extreme cases of shock, the patient is almost invariably of a grayish blue color.

In hemorrhage, a definite increase in the white blood count is found. In shock, there was no increase in the white blood count·

In acute hemorrhage transfusion of blood is specific. The effects generally are striking, immediate, and permanent.

In cases where there is both hemorrhage and shock, transfusion proved effective, but the results were not as good as those following transfusion for hemorrhage alone.

In shock without hemorrhage, if the patient had not yet reached the gray-blue stage, a certain amount of benefit followed transfusion of blood. If the patient had reached the gray-blue stage no benefit resulted from transfusion.

## Figures and Tables

**Figure f1:**